# Association of Human Q Fever with Animal Husbandry, Taiwan, 2004–2012

**DOI:** 10.3201/eid2112.141997

**Published:** 2015-12

**Authors:** Chung-Hsu Lai, Lin-Li Chang, Jiun-Nong Lin, Ming-Huei Liao, Shyh-Shyan Liu, Hsu-Hsun Lee, Hsi-Hsun Lin, Yen-Hsu Chen

**Affiliations:** Kaohsiung Medical University, Kaohsiung City, Taiwan (C.-H. Lai, L.-L. Chang, J.-N. Lin, Y.-H. Chen);; E-Da Hospital/I-Shou University, Kaohsiung City (C.-H. Lai, J.-N. Lin, H.-H. Lin);; National Pingtung University of Science and Technology, Neipu, Taiwan (M.-H. Liao, S.-S. Liu, H.-H. Lee);; National Yang-Ming University, Taipei City, Taiwan (H.-H. Lin):; National Chiao Tung University, HsinChu, Taiwan (Y.-H, Chen)

**Keywords:** Q fever, epidemiology, cattle, goat, goat pox, zoonoses, Taiwan, bacteria, Coxiella burnetii

## Abstract

In Taiwan, Q fever cases in humans began increasing in 2004 and peaked in 2007 but dramatically declined in 2008 and 2011. Cases were significantly correlated with the number of goats. The decline might be associated with the collateral effects of measures to control goat pox in 2008 and 2010.

Q fever is a zoonosis caused by infection with *Coxiella burnetii*, a gram-negative bacterium and obligate intracellular pathogen. The major animal reservoirs are goats, sheep, and cattle. Humans are infected mainly through inhalation of aerosolized particles contaminated with *C. burnetii* excreted by an infected animal, particularly in fetal products, milk, urine, and feces (*1*). Nearly 60% of primary infections (acute Q fever) are asymptomatic. Symptomatic infection is characterized by influenza-like symptoms, occasionally accompanied by hepatitis or pneumonia.

Historically, the largest outbreak of Q fever caused nearly 4,000 human infections in the Netherlands during 2007–2010 (*2*). Epidemiologic studies implicated infected farm animals (sheep and goats) as the source for human infection (*2*,*3*). In the United States, an outbreak was associated with fetal products from aborted goats (*4*). Because of improved recognition and reporting of Q fever, it became reportable in the United States in 1999, and the number of cases increased by 250% during 2000–2004 (*5*). 

In Taiwan, Q fever is endemic, particularly in the south (*6*). We previously identified the epidemiology and clinical characteristics of Q fever in Taiwan, but the association between human Q fever and animal husbandry has not been investigated (*7*–*9*). Our objective was to investigate the epidemiology of human Q fever and its association with animals by analyzing nationwide databases of human Q fever and animal husbandry during 2004–2012. 

## The Study

The Ethics Committee of E-Da Hospital (EMRP-103–042) approved this study. We collected data on confirmed human Q fever cases in Taiwan that occurred during 2004–2012 from the notifiable infectious diseases statistics system established by the Centers for Disease Control and Prevention of Taiwan (Taiwan CDC), which is an open and public website (http://nidss.cdc.gov.tw/ch/SingleDisease.aspx?dc=1&dt=4&disease=0830). The data included the number of confirmed cases, patient sex, age groups (5-year groups), and the geographic locations of cases (county and district) in Taiwan every month from 2004 through 2012. Reported cases included suspected cases of Q fever reported to the Taiwan CDC by clinicians. Generally, paired blood specimens (acute or convalescent phase) from reported case-patients are collected and sent to the contracted laboratories of the Taiwan CDC for laboratory testing of Q fever. Confirmed cases are reported cases that are confirmed positive for Q fever by laboratory tests. Q fever was confirmed either by serologic detection of a >4-fold increase in specific antibodies against *C. burnetii* phase II antigen by using an indirect immunofluorescence antibody assay or by a molecular method consisting of positive detection of *C. burnetii* DNA in blood using PCR.

We collected husbandry data on goats and cattle from 2004 through 2012 from the open and publicly available data released by the Council of Agriculture, Executive Yuan, Taiwan (http://agrstat.coa.gov.tw/sdweb/public/official/OfficialInformation.aspx). The maps of the geographic distributions of Q fever cases and animals were created by using SuperGIS Desktop software (Supergeo Technologies Inc., Taipei, Taiwan).

**Figure 1:**
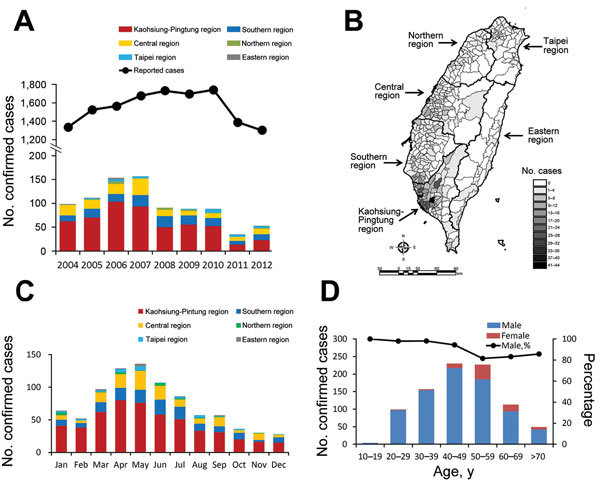
Q fever in humans, Taiwan, 2004–2012. A) Trends in reported and confirmed cases of Q fever. B) Geographic distribution of confirmed cases of Q fever. C) Monthly distribution of the confirmed cases. D) Age and sex distributions of patients with confirmed Q fever.

We identified 879 (6.3%) confirmed cases of Q fever among the 13,962 cases reported during 2004–2012. The number of confirmed cases increased dramatically starting in 2004 and peaked in 2007 but declined in 2008 and 2011 (Figure 1, panel A). Additionally, the annual incidence increased from 0.44 cases per 100,000 population in 2004 to 0.68 in 2007 and decreased from 0.40 in 2008 to 0.15 in 2011. Overall average annual incidence was 0.43 cases per 100,000 population. Cases occurred mainly in southern Taiwan (674 [76.7%] of 879) and particularly in the southern (17.4%) and Kaohsiung-Pingtung (59.3%) regions (Figure 1, panels A, B), and were most prevalent from March through September (669 [76.1%] cases) (Figure 1, panel C). Most case-patients were 30–69 years old (727 [82.7%]) and male (793 [90.2%]) (Figure 1, panel D).

**Figure 2:**
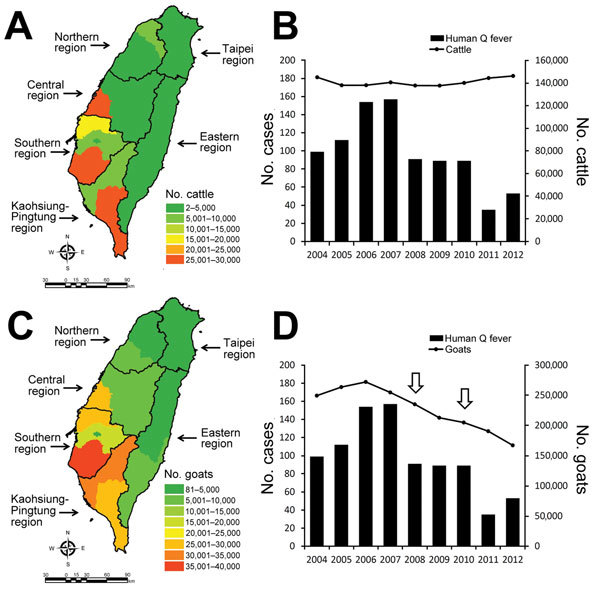
Q fever in cattle and goats and comparison with number of human Q fever cases, Taiwan, 2004–2012. A) Average number and distribution of cattle during 2004–2012; B) comparison of human Q fever cases and number of cattle showing no correlation (p = 0.123). C) Average number and distribution of goats during 2004–2012; D) comparison of human Q fever cases and the number of goats showing a significant correlation (p = 0.003). Arrows indicate goat pox epidemics of 2008 and 2010. The correlation between human Q fever, cattle, and goat was analyzed by Pearson’s correlation.

During 2004–2012, cattle and goats were distributed primarily in the southern and Kaohsiung-Pingtung regions (Figure 2, panels A, C). The trend of human Q fever cases was significantly correlated with the number of goats, rather than with the number of cattle (Figure 2, panels B, D).

## Conclusions

During 2004–2012, the average incidence of Q fever in Taiwan was 0.43 cases per 100,000 population, which was higher than the incidence in the United States (0.04) (*10*) but lower than that in France (2.5) (*11*). Regardless of these differences in incidence, Q fever cases increased after Q fever became notifiable in the United States (*5*,*10*) and France (*11*). This increase might be attributed to improved recognition and increased reporting of notifiable infectious diseases to the authorities. In Taiwan, we have previously illustrated that reported and confirmed Q fever cases dramatically increased beginning in 2004, 3 years before it became notifiable in October 2007 (*6*). However, confirmed cases of Q fever decreased in 2008, even though the number of reported cases remained steady during 2007–2010 (Figure 1, panel A). Accordingly, the changing number of confirmed cases could not be explained by a change in the number of reported cases or by the advent of Q fever as a notifiable disease in Taiwan.

Most cases occurred in southern Taiwan, particularly in the Kaohsiung-Pingtung region, despite fluctuations in the yearly number of cases (Figure 1, panels A, B). Geographic distribution was correlated with the distribution of cattle and goat husbandry, which was predominant in southern Taiwan (Figure 2, panels A, C). A serologic study in the Kaohsiung-Pingtung region found a high seroprevalence of Q fever in animals (*12*). Seroprevalence rates in the overall herd and in individual animals were, respectively, 73.6% and 48.3% in goats and 66.7% and 19.5% in cattle. In addition, the 26.3% seroprevalence in persons engaging in veterinary and animal-related work was higher than in the reference population (2.7%). Thus, we suspected that the decrease in the number of human cases might have been associated with animal reservoirs, particularly goats.

Goats and cattle are the major animal reservoirs of *C. burnetii*. We illustrated that the increase and decrease in human Q fever cases was correlated with variation in the number of goats, rather than cattle (Figure 2, panel B). After the number of goats began to decrease in 2007, human Q fever cases dramatically decreased in 2008 and 2011. The decrease in the number of goats was possibly associated with 2 episodes of goat pox epidemics in July 2008 and April 2010 (Figure 2, panel D) and the culling of 210 and >20,000 goats in the 2008 and 2010 epidemics, respectively (*13*,*14*). To control the goat pox epidemics, several measures were enacted, including animal and vehicle movement control within infected areas, cleaning and disinfection of infected farms and equipment, culling of infected animals, and vaccination. Although these measures were applied to control goat pox, collateral effects that diminished the spread of *C. burnetii* from infected goats or a contaminated environment to humans also might have existed.

This study has certain limitations. The association between livestock numbers and human Q fever might be ecologic because data on individual exposures and on Q fever in goats and cattle over time were not available for analysis.

In conclusion, Q fever is an endemic disease in Taiwan. Human cases increased beginning in 2004 and decreased in 2008 and 2011, which was correlated with the number of goats and possibly was associated with the collateral effects of measures taken to control goat pox in 2008 and 2010.
